# TRIM58 Interacts with Pyruvate Kinase M2 to Inhibit Tumorigenicity in Human Osteosarcoma Cells

**DOI:** 10.1155/2020/8450606

**Published:** 2020-03-07

**Authors:** Peng Yuan, Yiyi Zhou, Rui Wang, Shayang Chen, Qiqi Wang, Zhujie Xu, Yi Liu, Huilin Yang

**Affiliations:** ^1^Department of Orthopaedics, The Affiliated Wuxi People's Hospital of Nanjing Medical University, Wuxi 214000, China; ^2^Department of Orthopaedic Surgery, The First Affiliated Hospital of Soochow University, Suzhou 215006, China

## Abstract

**Background:**

Tripartite motif containing 58 (TRIM58), an E3 ubiquitin ligase, is reported as a suppressor gene in certain human tumors. However, the biological function of TRIM58 in osteosarcoma (OS) is still less identified.

**Methods:**

In the present study, TRIM58 induced silencing and overexpression in OS cells using RNA interference (RNAi) and lentiviral-mediated vector, respectively. Cell proliferation profiles were analyzed using cell counting kit-8 (CCK-8) assay. Cell apoptosis profiles were determined using a flow cytometer. qRT-PCR and western blot were used to determine gene expression. Coimmunoprecipitation (Co-IP) assay was used to examine protein interaction.

**Results:**

Our results demonstrated TRIM58 was downregulated in human OS tissues. Overexpression of TRIM58 remarkably suppressed the growth of OS cells and decreased glucose transportation and lactate secretion. These results indicated that TRIM58 involved in the regulation of energy metabolism in OS cells. Importantly, TRIM58 interacted with pyruvate kinase M2 (PKM2) in OS cells. Moreover, TRIM58 might inhibit the activity of PKM2 through enhancing its polyubiquitination in OS cells.

**Conclusions:**

This analysis not only explored a deep understanding of the biological function of TRIM58 but also indicated its signaling pathway in OS cells.

## 1. Introduction

Osteosarcoma (OS) is one of the primary malignant tumors, which is caused by malignant osteoid production and osteoblastic differentiation [1]. OS is commonly identified in adolescence, and incidence significantly varies with age [2]. Although chemotherapy has contributed to the treatment of OS, the outcome is far from satisfactory. Therefore, the novel effective therapeutics are urgently needed.

Tripartite motif containing 58 (TRIM58) belongs to the tripartite motif-containing family, which possess E3 ubiquitin ligase activities [3]. Previous report has demonstrated that TRIM58 regulates terminal erythropoiesis [4]. Moreover, TRIM58 is reported as a potential biomarker for colorectal cancer and identified as a tumor suppressor gene [5]. Further, it has been confirmed that TRIM58 is significantly downregulated in hepatocellular carcinoma tissues [6]. Furthermore, TRIM58 has enhanced the degradation of dynein holoprotein complex through ubiquitination [4]. However, the precise biological function of TRIM58 is still not clear in OS.

It has been well described that glucose can be used as a carbon source for aerobic glycolysis in cancer cells, which is known as the Warburg effect [7]. Moreover, the activity of glycolysis is commonly increased in cancer cells due to its extra energy consumption. Glycolysis inhibition is reported as a promising approach for cancer treatment [8].

Tumor-specific pyruvate kinase M2 (PKM2) is an isozyme of pyruvate kinase, which contributes to the Warburg effect and gene transcription [9, 10]. Moreover, previous reports have indicated that PKM2 is upregulated in human cancers [11, 12]. The activity of PKM2 has been mediated by posttranslational modification. Previous report has demonstrated that TRIM35 inhibits the phosphorylation of PKM2 in hepatocellular carcinoma cells and reduces its tumorigenicity [13]. Moreover, carboxyl terminus of Hsc70-interacting protein has decreased the activity of PKM2 through enhancing its ubiquitination in ovarian carcinoma cells [14]. Therefore, suppressing the activity of PKM2 is a promising approach in the treatment of human cancers [15]. However, the molecule network of PKM2 is still less identified in OS cells.

In the current study, TRIM58 induced silencing by RNA interference (RNAi) and overexpression by lentiviral vector in OS cell lines. Our analysis not only elucidated the role of TRIM58 in the pathogenesis of OS cells but also indicated its potential target in OS.

## 2. Materials and Methods

### 2.1. OS Samples and Cell Lines

A total of 20 human OS tumor and 12 normal bone tissues were used to explore the expression of TRIM58. Moreover, all the cell lines involved in this research were purchased from the cell bank of the Shanghai Biology Institute (Shanghai, China), including U2OS, SAOS2, MG63, HOS, 143B, and hFOB1.19. Cells were cultured in a 5% CO_2_ atmosphere at 37°C. The inhibitor MG132 (Selleck, USA) was dissolved in DMSO. This research was approved by the Ethics Committee of the First Affiliated Hospital of Soochow University and followed the tenants of the Declaration of Helsinki.

### 2.2. RNA Extraction and qRT-PCR

Total RNA was extracted by using TRIzol reagent (1596-026, Invitrogen, USA). Then, complementary DNA (cDNA) was synthesized from extracted RNA with the cDNA synthesis kit (#K1622, Fermentas, Canada) according to the instructions of the manufacturer. The expression of GAPDH was applied as the reference gene and counted by using the 2^−ΔΔCt^ method. Three replicates were needed for each analysis. The primers used in this study are presented in Supplementary [Supplementary-material supplementary-material-1].

### 2.3. Lentiviral-Mediated Silenced and Overexpression of TRIM58

TRIM58 (NM_015431.3) siRNAs (siTRIM58) and nonspecific scrambled siRNA (siNC) were specifically designed and synthesized (Major, China). A lentiviral plasmid (pLVX-puro) contains the full length of the human TRIM58 cDNA sequence. A mock plasmid functioned as negative controls (oeNC). Lipofectamine 2000 (Invitrogen, USA) was used to transiently transfected plasmids into cells, respectively. The sequences of siTRIM58s are provided in Supplementary [Supplementary-material supplementary-material-1].

### 2.4. Western Blot

Cells were lysed using RIPA lysis buffer (JRDUN, Shanghai, China) containing EDTA-free protease inhibitor cocktail (Roche, Germany). BCA protein assay kit (Thermo Fisher, USA) was utilized to estimate the protein content. Equal amounts of protein (25 *μ*g) were separated using 10% SDS-PAGE and transferred to a nitrocellulose membrane (Millipore, USA) overnight. After that, the membranes were blocked with 5% nonfat dry milk for 1 h at room temperature and then probed overnight at 4°C with primary antibodies. Next, the membrane was washed three times with TBST and probed with the secondary antibody (anti-mouse IgG) for 1 h at 37°C. An enhanced chemiluminescence system (Tanon, China) was used to detect the protein level. Three replicates were needed for each sample, and GAPDH was used as the internal reference. The information of primary antibodies is listed in Supplementary [Supplementary-material supplementary-material-1].

### 2.5. Cell Proliferation Assay

Cell proliferation profile was analyzed by using cell counting kit-8 (CCK-8) assay (CP002, SAB, USA) according to the protocol of the manufacturer. The optical density values (OD) at a wavelength of 450 nm were examined by a microplate reader (DNM-9602, Pulangxin, China). Three replicates were needed for each sample.

### 2.6. Cell Apoptosis Assay

Cell apoptosis profile was determined by using Annexin V-fluorescein isothiocyanate (FITC) apoptosis detection kit (C1063, Beyotime, China) according to the instructions of the manufacturer. Flow cytometer (Accuri C6, BD, USA) was used to determine cells at 48 h after viral infection.

### 2.7. Glucose Transport and Lactate Production Assay

In brief, the glucose analog 2-NBDG was used as a fluorescent probe for determining the activity of glucose transport. A total of 5 × 105 cells from different groups were seeded in 6-well plates. Then, all the cells were preincubated in Krebs-Ringer bicarbonate (KRB) buffer (glucose-free) for 15 min after maintaining in a 5% CO_2_ atmosphere at 37°C for 24 h. After that, cells were incubated in fresh KRB buffer supplemented with 2-NBDG for 45 min at 37°C, 5% CO_2_. Flow cytometry using a GloMax®-Multi + flow cytometer (Promega, USA) was used to quantitatively analyze the stained cells. Moreover, Lactate Assay Kit (Njjcbio, China) was used to analyze the production of lactate in different cells according to the manufacturer's protocol.

### 2.8. Coimmunoprecipitation (Co-IP)

For IP, whole-cell extracts were prepared after transfection or stimulation with appropriate ligands, followed by incubation overnight with the appropriate antibodies plus protein A/G beads. Beads were washed five times and separated by SDS-PAGE. Western blot was performed using the antibodies as indicated.

### 2.9. Ubiquitination Assay

MG63 cells that transfected with oeTRIM58 were lysed in 1% SDS-containing radio immunoprecipitation assay (RIPA) buffer by sonication on ice. Then, lysates were treated with Protein A/G Plus-Agarose (sc-2003, Santa Cruz, USA) for 1 h. After that, each sample was incubated with the IgG (30000-0-AP, Proteintech, USA) overnight at 4°C. Then, the nuclear pellet was collected by centrifugation at 3000 rpm for 5 min at 4°C and subsequently washed four times by Protein A/G Plus-Agarose beads. The purified proteins were separated by gradient SDS-PAGE. Anti-PKM2 (ab137852, Abcam, UK) or anti-ubiquitin antibody (ab7780, Abcam, UK) was used for immunoblotting.

### 2.10. Statistical Analysis

Statistical analyses were performed by using GraphPad Prism software version 7.0 (CA, USA). Data were presented as the mean ± SD. Statistical significance was determined by one-way analysis of variance (ANOVA) for multiple comparisons. Significance was set at *p* value<0.05.

## 3. Results

### 3.1. TRIM58 Was Downregulated in OS Tissues

**Figure 1:**
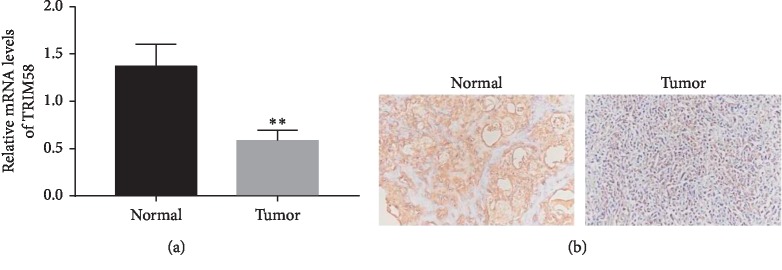
TRIM58 was downregulated in OS tissues. (a) The mRNA level of TRIM58 was detected in human OS tissues and normal bone samples. ^*∗∗*^*p* < 0.01 vs. normal. (b) The protein levels of TRIM58 in human OS tissues and normal bone samples were examined by immunohistochemistry assay.

In this study, we examined the relative mRNA levels of TRIM58 in human OS (*n* = 20) and normal bone tissues (*n* = 12), respectively. Clearly, the relative mRNA level of TRIM58 was deeply decreased in human OS tissues compared with that in normal matched bone samples (Figure 1(a)).

Moreover, immunohistochemistry assay was established to detect the protein content of TRIM58 in OS tissues. Interestingly, the protein content of TRIM58 was also deeply inhibited in OS tissues (Figure 1(b)). Taken together, all these results indicated that TRIM58 was downregulated in OS tissues and cells.

### 3.2. Silencing and Overexpression of TRIM58 in OS Cells

**Figure 2:**
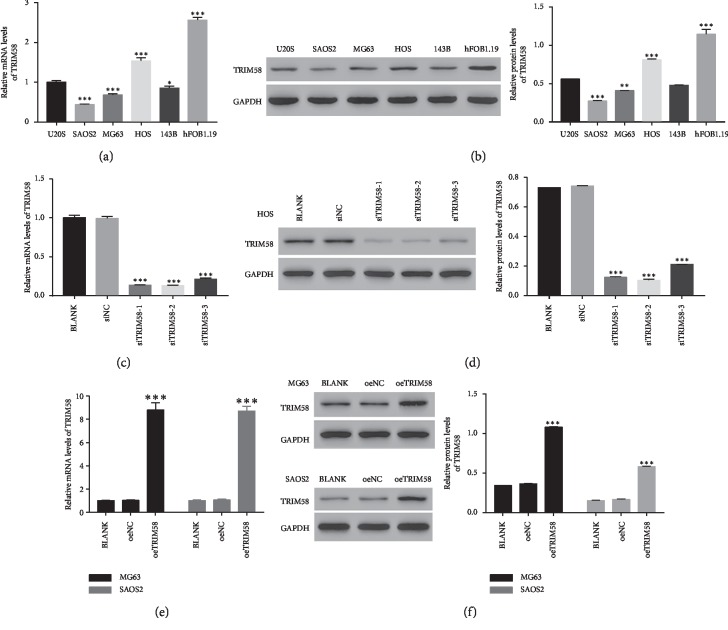
Knockdown and overexpression of TRIM58 in OS cells. (a, b) The relative mRNA and protein levels of TRIM58 in different OS cells (U2OS, SAOS2, MG63, HOS, and 143B) and normal osteoblastic cells (hFob1.19). ^*∗*^*p* < 0.05 vs. U2OS; ^*∗∗*^*p* < 0.01 vs. U2OS; ^*∗∗∗*^*p* < 0.001 vs. U2OS. (c, d) The relative mRNA and protein levels of TRIM58 in siTRIM58-transfected cells. ^*∗∗∗*^*p* < 0.001 vs. BLANK. (e, f) The relative mRNA and protein levels of TRIM58 in oeTRIM58-transfected cells. ^*∗∗∗*^*p* < 0.001 vs. BLANK.

Next, we quantify the levels of TRIM58 in five human OS cell lines, including U2OS, SAOS2, MG63, HOS, and 143B and normal osteoblastic cells (hFob1.19) (Figures 2(a) and 2(b)). Both the relative mRNA and protein contents of TRIM58 were extremely decreased in OS cells, especially in SAOS2 and MG63 cells. Therefore, TRIM58 overexpression was induced in these two cell lines. Meanwhile, knockdown of TRIM58 was established in HOS cells.

Three small short interfering RNAs targeting the human TRIM58 gene (siTRIM58-1, siTRIM58-2, and siTRIM58-3) were synthesized. Meanwhile, a nonspecific siRNA was served as a negative control (siNC). Then, all of them were transfected into HOS cells, respectively. Clearly, the relative mRNA and protein levels of TRIM58 were deeply abolished by siTRIM58 (Figures 2(c) and 2(d)). Moreover, our results indicated that siTRIM58-1 and siTRIM58-2 presented a stronger effect than siTRIM58-3. Meanwhile, a plasmid for overexpressing TRIM58 (oeTRIM58) and mock plasmid (oeNC) were generated and subsequently transfected into MG63 and SAOS2 cells, respectively. Obviously, oeTRIM58 significantly improved the expression of TRIM58 in oeTRIM58-transfected cells in two cell lines (Figures 2(e) and 2(f)). Hence, the siTRIM58-1-, siTRIM58-2-, and oeTRIM58-transfected cells were used in the following analyses.

### 3.3. Overexpression of TRIM58 Inhibited the Growth of OS Cells

**Figure 3:**
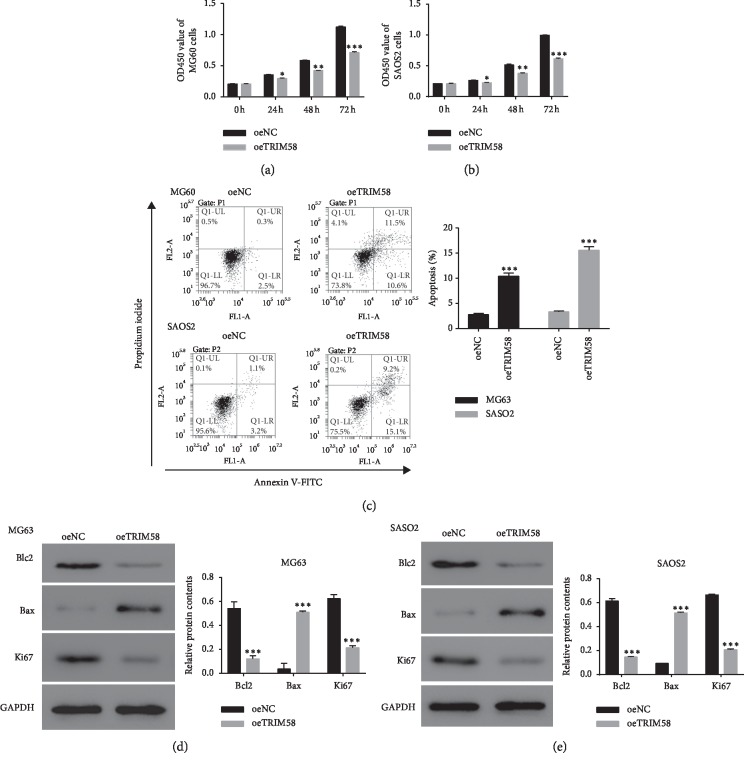
Overexpression of TRIM58 suppressed the progression of OS cells. (a, b) The cell proliferation profile of MG60 and SAOS2 cells transfected with oeTRIM58, respectively. ^*∗*^*p* < 0.05 vs. oeNC; ^*∗∗*^*p* < 0.01 vs. oeNC; ^*∗∗∗*^*p* < 0.001 vs. oeNC. (c) The cell apoptosis profile of MG60 and SAOS2 cells transfected with oeTRIM58, respectively. ^*∗∗∗*^*p* < 0.001 vs. oeNC. (d, e) The protein contents of Bcl2, Bax, and Ki67 were examined in MG60 and SAOS2 cells transfected with oeTRIM58, respectively. ^*∗∗∗*^*p* < 0.001 vs. oeNC.

We also examined the function of oeTRIM58 in OS cells. As shown in Figures 3(a) and 3(b), the cell proliferation profile was deeply decreased in oeTRIM58-transfected cells in two cell lines. Moreover, overexpression of TRIM58 obviously improved the apoptosis of OS cells (Figure 3(c)).

B cell lymphoma 2 (Bcl2) is widely recognized as the antiapoptosis, whereas the Bax is reported as a proapoptosis factor [16, 17]. In the current study, TRIM58 overexpression significantly suppressed the translation of Bcl2 in MG63 and SASO2 cells, but promoted the expression of Bax. Moreover, Ki67, the representative proliferation marker [18], was also downregulated in MG63 and SASO2 cells that transfected with oeTRIM58 (Figures 3(d) and 3(e)). Overall, these results indicated that overexpression of TRIM58 inhibited the growth of OS cells.

### 3.4. The Glycolysis Activity Was Inhibited in oeTRIM58-Transfected Cells

**Figure 4:**
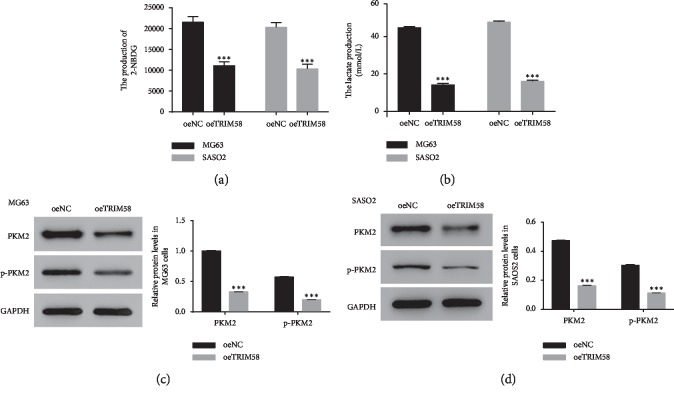
TRIM58 overexpression suppressed the glycolysis activity in human OS cells. (a, b) The production of 2-NBDG and lactate in MG60 and SAOS2 cells transfected with oeTRIM58, respectively. ^*∗∗∗*^*p* < 0.001 vs. oeNC. (c, d) The protein level of PKM2 and p-PKM2 in MG60 and SAOS2 cells transfected with oeTRIM58, respectively. ^*∗∗∗*^*p* < 0.001 vs. oeNC.

Next, we examined the function of TRIM58 in glycolysis metabolism. Our results suggested that the production of 2-NBDG and lactate was deeply downregulated in oeTRIM58-transfected cells (Figures 4(a) and 4(b)). We also quantified the protein content of PKM2 and p-PKM2 in oeTRIM58-transfected cells in two cell lines. As shown in Figures 4(c) and 4(d), the protein levels of PKM2 and p-PKM2 were significantly decreased in oeTRIM58-transfected cells. Therefore, overexpression of TRIM58 inhibited the growth of OS cells and involved in PKM2 signaling pathway in OS cells.

### 3.5. Knockdown of TRIM58 Promoted the Progression of OS Cells

**Figure 5:**
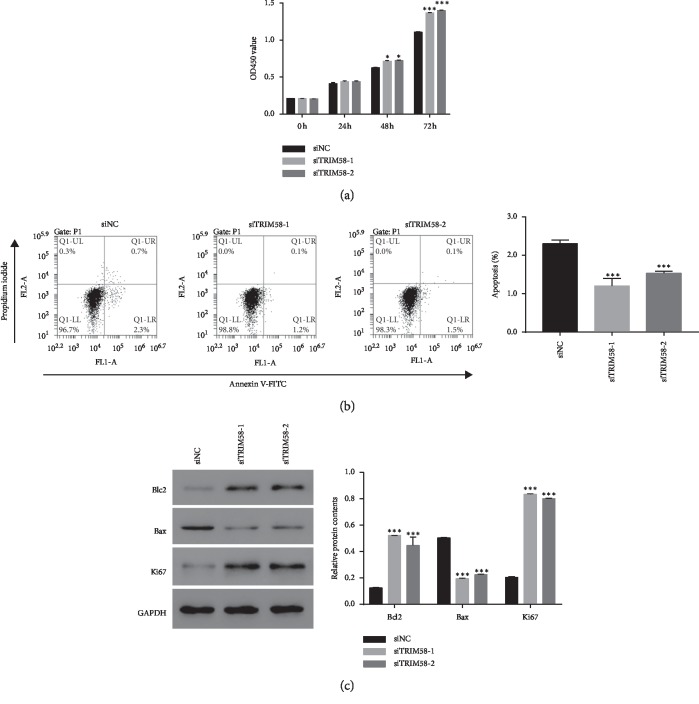
TRIM58 silencing promoted the progression of human OS cells. (a) The cell proliferation of siTRIM58-transfected cells was detected in 0, 24, 48, and 72 h. ^*∗*^*p* < 0.05 vs. U2OS; ^*∗∗∗*^*p* < 0.001 vs. siNC. (b) The cell apoptosis rate was inhibited by siTRIM58. ^*∗∗∗*^*p* < 0.001 vs. siNC. (c) The protein contents of Bcl2, Bax, and Ki67 were examined in U2OS cells transfected with siTRIM58, respectively. ^*∗∗∗*^*p* < 0.001 vs. siNC.

Next, the cell proliferation rate of siTRIM58-transfected cells was quantified using cell counting kit-8 (CCK-8) assay. As shown in Figure 5(a), the proliferation rate was remarkably upregulated in siTRIM58-1 or siTRIM58-2-transfected cells. Moreover, the apoptosis rates of TRIM58 siRNA-transfected cells were much lower than those of siNC-transfected cells (Figure 5(b)). Further, the content of Bcl2 was significantly upregulated in siTRIM58-1- or siTRIM58-2-transfected cells, while the level of Bax was opposite to that of Bcl2. Furthermore, knockdown of TRIM58 significantly promoted the translation of Ki67 in OS cells. Taken together, TRIM58 silencing contributed to the growth of OS cells.

### 3.6. TRIM58 Silencing Promoted Lactate Production and Glucose Transport Activity in Human OS Cells

**Figure 6:**
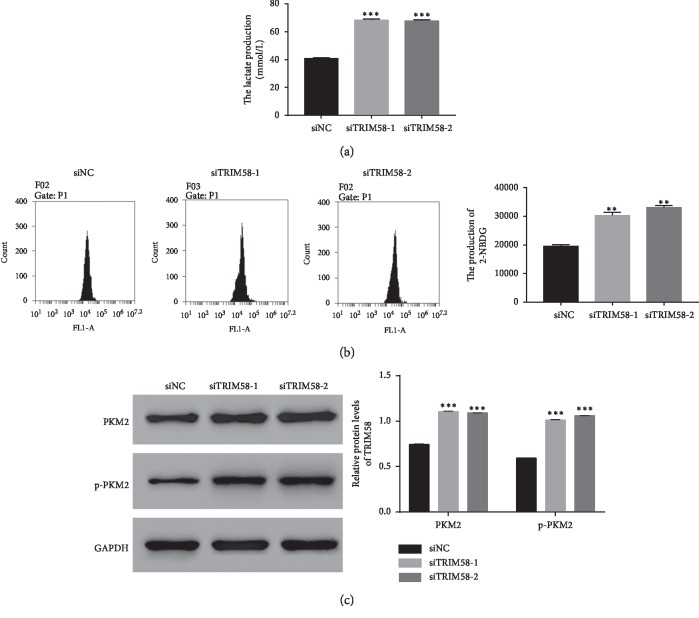
TRIM58 silencing promoted lactate production and glucose transport activity in human OS cells. (a) The lactate production was increased in siTRIM58-transfected cells. ^*∗∗∗*^*p* < 0.001 vs. siNC. (b) The production of 2-NBDG was increased in siTRIM58-transfected cells. ^*∗∗*^*p* < 0.01 vs. siNC. (c) The protein content of PKM2 and p-PKM2 was upregulated in siTRIM58-transfected cells. ^*∗∗∗*^*p* < 0.001 vs. siNC.

Next, we examined the lactate production in siTRIM58-transfected cells. Obviously, the production of lactate was much higher in siTRIM58-transfected cells than that in siNC-transfected cells (Figure 6(a)). Moreover, the glucose analog 2-NBDG was used to examine the glucose transport activity. Our result has indicated that the production of 2-NBDG was increased in siTRIM58-transfected cells. Therefore, TRIM58 inhibited the production of lactate and the transportation of glucose in OS cells (Figure 6(b)). Interestingly, the protein contents of both PKM2 and p-PKM2 were increased in siTRIM58-1-transfected cells (Figure 6(c)). Taken together, all these results demonstrated that TRIM58 involved in PKM2 signaling pathway in human OS cells.

### 3.7. TRIM58 Interacted with PKM2 and Enhanced Its Ubiquitination in OS Cells

**Figure 7:**
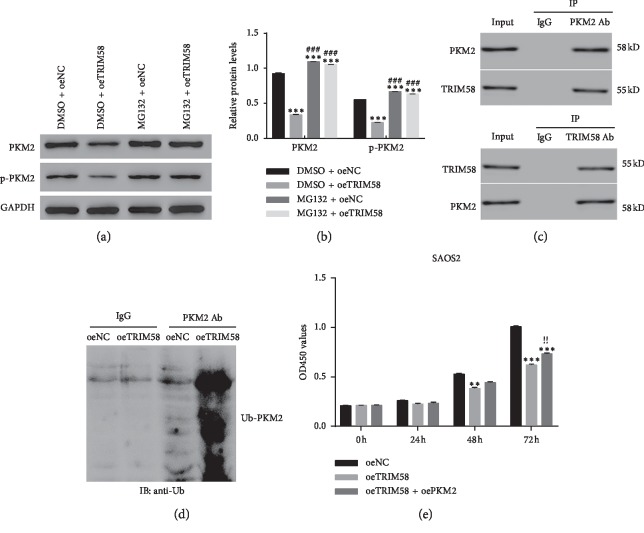
TRIM58 interacted with PKM2 and enhanced its polyubiquitination in OS cells. (a, b) The relative protein contents of PKM2 and p-PKM2 were inhibited by MG132 in oeTRIM58-transfected cells. ^*∗∗∗*^*p* < 0.001 vs. DMSO + oeNC. ^###^*p* < 0.001 vs. DMSO + oeTRIM58. (c) Co-IP assay was performed to examine the interaction between TRIM58 and PKM2 in OS cells. (d) Overexpression of TRIM58 enhanced the polyubiquitination of PKM2 in OS cells. (e) PKM2 overexpression promoted the proliferation of oeTRIM58-transfected cells. ^*∗∗*^*p* < 0.01 vs. oeNC. ^*∗∗∗*^*p* < 0.001 vs. oeNC; ^!!^*p* < 0.01 vs. oeTRIM58.

A specific ubiquitin-proteasome inhibitor MG132 was used to block the endogenous ubiquitin activity in OS cells. As shown in Figures 7(a) and 7(b), it was easily identified that PKM2 and p-PKM2 were significantly downregulated in oeTRIM58-transfected cells, whereas this effect was deeply abolished by the inhibitor MG132.

Next, coimmunoprecipitation (CO-IP) assay was performed to further examine the correlation between TRIM58 and PKM2 in OS cells. Clearly, there was a stronger interaction between TRIM58 and PKM2 in OS cells (Figure 7(c)). Moreover, intracellular ubiquitination assays were established to examine whether TRIM58 affected the polyubiquitination of PKM2. As shown in Figure 7(d), the polyubiquitination activity of PKM2 was much higher in oeTRIM58-transfected cells than that of oeNC-transfected cells. Taken together, all these results demonstrated that TRIM58 interacted with PKM2 and enhanced its ubiquitination in OS cells. More importantly, PKM2 overexpression promoted the proliferation of oeTRIM58-transfected cells (Figure 7(e)). These findings elucidated that overexpression of PKM2 abolished the function of TRIM58 in OS cells. TRIM58 might suppress the progression of OS cells by targeting PKM2.

## 4. Discussion

OS has ranked second place in the most common primary malignant bone tumors, which is commonly occurred in children and adolescents [19]. However, the outcome of traditional therapies (including surgery, radiotherapy, and systemic therapy) is not sufficient [20, 21]. Therefore, the effective target is a critical step to develop a novel approach for OS therapy. In this analysis, we found TRIM58 was downregulated in OS tissues and cells. Moreover, our results indicated that TRIM58 was a suppressor gene in OS cells. Therefore, our findings provided novel insight into the treatment for OS.

The progression of cancer often consumes extra energy, which has accelerated the activity of ATP transportation. Previous report has indicated that the glycolysis metabolism is the main energy source for cancer cells [8]. Moreover, the activity of glycolysis is closely associated with the growth of OS cells [22, 23]. In this study, TRIM58 overexpression decreased the glucose consumption and lactate secretion of OS cells. These results indicated that TRIM58 suppressed the activity of glycolysis in OS cells. Moreover, similar results were also obtained in siTRIM58-transfected cells. Therefore, our findings suggested that TRIM58 might inhibit the progression of OS cells through regulating energy transportation.

It has been confirmed that PKM2 plays a key role in energy metabolism, which catalyzes the last step of glycolysis [24, 25]. Moreover, growing evidences have demonstrated that PKM2 is a central factor in transcription and tumorigenesis and often upregulated in certain human cancers [26, 27]. Further, a previous report has indicated that TRIM35 interacts with PKM2 and suppressed the tumorigenicity in hepatocellular cancer [13]. In the present study, we found TRIM58 overexpression inhibited the translation of PKM2 and enhanced its ubiquitination in human OS cells. Hence, our findings demonstrated that TRIM58 disrupted the progression of OS cells by inhibiting the activity of PKM2. Targeting TRIM58/PKM2 pathway presented novel insight into the treatment for OS.

## 5. Conclusion

In the present study, our results indicated that TRIM58 was an antiproliferation and proapoptosis factor in OS cells. Moreover, TRIM58 involved in the regulation of lactate production and glucose transport activity in OS cells. Importantly, TRIM58 interacted with PKM2 and enhanced its ubiquitination in OS cells. In total, present research not only demonstrated the underlying function of TRIM58 in OS cells but also indicated its signaling pathway as well as provided novel insight in developing OS therapy approach.

## Data Availability

All data generated or analyzed during this study are included in this published article.

## Supplementary Materials

Supplementary Materials Supplementary File 1: primer sequence information. Supplementary Table 1: human gene TRIM58 (NM_015431.3) RNAi targeting locus information. Supplementary Table 2: the primary antibody information.

## References

[1] Ottaviani G., Jaffe N. (2009). The epidemiology of osteosarcoma. *Cancer Treatment and Research*.

[2] Kager L., Zoubek A., Dominkus M. (2010). Osteosarcoma in very young children. *Cancer*.

[3] Napolitano L. M., Meroni G. (2012). TRIM family: pleiotropy and diversification through homomultimer and heteromultimer formation. *IUBMB Life*.

[4] Thom C. S., Traxler E. A., Khandros E. (2014). Trim58 degrades dynein and regulates terminal erythropoiesis. *Developmental Cell*.

[5] Liu M., Zhang X., Cai J. (2018). Downregulation of TRIM58 expression is associated with a poor patient outcome and enhances colorectal cancer cell invasion. *Oncology Reports*.

[6] Qiu X., Huang Y., Zhou Y., Zheng F. (2016). Aberrant methylation of TRIM58 in hepatocellular carcinoma and its potential clinical implication. *Oncology Reports*.

[7] Song J., Wu X., Liu F. (2017). Long non-coding RNA PVT1 promotes glycolysis and tumor progression by regulating miR-497/HK2 axis in osteosarcoma. *Biochemical and Biophysical Research Communications*.

[8] Gill K. S., Fernandes P., O’Donovan T. R. (2016). Glycolysis inhibition as a cancer treatment and its role in an anti-tumour immune response. *Biochimica et Biophysica Acta (BBA)-Reviews on Cancer*.

[9] Jiang Y., Li X., Yang W. (2014). PKM2 regulates chromosome segregation and mitosis progression of tumor cells. *Molecular Cell*.

[10] Koppenol W. H., Bounds P. L., Dang C. V. (2011). Otto Warburg’s contributions to current concepts of cancer metabolism. *Nature Reviews Cancer*.

[11] Wong N., Melo J. D., Tang D. (2013). PKM2, a central point of regulation in cancer metabolism. *International Journal of Cell Biology*.

[12] Chaneton B., Gottlieb E. (2012). Rocking cell metabolism: revised functions of the key glycolytic regulator PKM2 in cancer. *Trends in Biochemical Sciences*.

[13] Chen Z., Wang Z., Guo W. (2015). TRIM35 interacts with pyruvate kinase isoform M2 to suppress the Warburg effect and tumorigenicity in hepatocellular carcinoma. *Oncogene*.

[14] Shang Y., He J., Wang Y. (2017). CHIP/Stub1 regulates the Warburg effect by promoting degradation of PKM2 in ovarian carcinoma. *Oncogene*.

[15] Li Y. H., Li X. F., Liu J. T. (2018). PKM2, a potential target for regulating cancer. *Gene*.

[16] Cui J., Placzek W. J. (2018). Post-transcriptional regulation of anti-apoptotic BCL2 family members. *International Journal of Molecular Sciences*.

[17] Porebska I., Wyrodek E., Kosacka M., Adamiak J., Jankowska R., Harlozinska-Szmyrka A. (2006). Apoptotic markers p53, Bcl-2 and Bax in primary lung cancer. *In Vivo*.

[18] Juríková M., Danihel Ľ., Polák Š., Varga I. (2016). Ki67, PCNA, and MCM proteins: markers of proliferation in the diagnosis of breast cancer. *Acta Histochemica*.

[19] Marina N., Gebhardt M., Teot L., Gorlick R. (2004). Biology and therapeutic advances for pediatric osteosarcoma. *The Oncologist*.

[20] Meyers P. A., Gorlick R. (1997). Osteosarcoma. *Pediatric Clinics of North America*.

[21] Ritter J., Bielack S. S. (2010). Osteosarcoma. *Annals of Oncology*.

[22] Yuan G., Zhao Y., Wu D., Gao C. (2017). Mir-150 up-regulates Glut1 and increases glycolysis in osteosarcoma cells. *Asian Pacific Journal of Cancer Prevention: APJCP*.

[23] Han X., Yang Y., Sun Y., Qin L., Yang Y. (2018). LncRNA TUG1 affects cell viability by regulating glycolysis in osteosarcoma cells. *Gene*.

[24] Dombrauckas J. D., Santarsiero B. D., Mesecar A. D. (2005). Structural basis for tumor pyruvate kinase M2 allosteric regulation and catalysis. *Biochemistry*.

[25] Liu F., Ma F., Wang Y. (2017). PKM2 methylation by CARM1 activates aerobic glycolysis to promote tumorigenesis. *Nature Cell Biology*.

[26] Yang W., Xia Y., Hawke D. (2012). PKM2 phosphorylates histone H3 and promotes gene transcription and tumorigenesis. *Cell*.

[27] Lee J., Kim H. K., Han Y.-M., Kim J. (2008). Pyruvate kinase isozyme type M2 (PKM2) interacts and cooperates with Oct-4 in regulating transcription. *The International Journal of Biochemistry & Cell Biology*.

