# Supplementary material for: TRIM58 Interacts with Pyruvate Kinase M2 to Inhibit Tumorigenicity in Human Osteosarcoma Cells
Source: Biomed Res Int. 2020 Mar 7;2020:8450606. doi: 10.1155/2020/8450606 (PMC7081029; doi:10.1155/2020/8450606)
Supplement: Supplementary Materials — Supplementary File 1: primer sequence information. Supplementary Table 1: human gene TRIM58 (NM_015431.3) RNAi targeting locus information. Supplementary Table 2: the primary antibody information. [file 8450606.f1.zip › 8450606.f1/Supplementary File1.pdf]

Supplementary File1: Primer sequence information

1.1 Homo sapiens tripartite motif containing 58 (TRIM58), mRNA

NM_015431.3

Primer F 5' CAACCCTGAGCGATTTGAC 3'

Primer R 5' TGGAGAAGAGGCACTGATG 3'

Pos: 972-1211

Amplified product: Size: 240 bps

1.2 Homo sapiens glyceraldehyde-3-phosphate dehydrogenase (GAPDH), transcript variant 2, mRNA

NM_001256799.2

Primer F 5' AATCCCATCACCATCTTC 3'

Primer R 5' AGGCTGTTGTCATACTTC 3'

Pos: 436-653

Amplified product: Size: 218 bps
