# Supplementary material for: TRIM58 Interacts with Pyruvate Kinase M2 to Inhibit Tumorigenicity in Human Osteosarcoma Cells
Source: Biomed Res Int. 2020 Mar 7;2020:8450606. doi: 10.1155/2020/8450606 (PMC7081029; doi:10.1155/2020/8450606)
Supplement: Supplementary Materials — Supplementary File 1: primer sequence information. Supplementary Table 1: human gene TRIM58 (NM_015431.3) RNAi targeting locus information. Supplementary Table 2: the primary antibody information. [file 8450606.f1.zip › 8450606.f1/Supplementary Table 2.pdf]

Supplementary Table 2: The primary antibodies information

Antibody name	Source	Dilution factor
TRIM58	Abcam, UK	1:500
PKM2	Abcam, UK	1:1000
Phospho-PKM2	Abcam, UK	1:500
Blc2	Abcam, UK	1:1000
Bax	Abcam, UK	1： 1000
Ki67	Abcam, UK	1:5000
GAPDH	CST, USA	1:2000
