# Supplementary material for: TRIM58 Interacts with Pyruvate Kinase M2 to Inhibit Tumorigenicity in Human Osteosarcoma Cells
Source: Biomed Res Int. 2020 Mar 7;2020:8450606. doi: 10.1155/2020/8450606 (PMC7081029; doi:10.1155/2020/8450606)
Supplement: Supplementary Materials — Supplementary File 1: primer sequence information. Supplementary Table 1: human gene TRIM58 (NM_015431.3) RNAi targeting locus information. Supplementary Table 2: the primary antibody information. [file 8450606.f1.zip › 8450606.f1/Supplementary Table1.pdf]

Supplementary Table1: Human gene TRIM58 (NM_015431.3) RNAi targeting locus information

RNAi Targeting Locus		Sequence
Name	locus positon	
siTRIM58-1	893-911	GGAGGGAGCTCTTAAGGAA
siTRIM58-2	1293-1311	GGACTATGAAGCCGGTGAA
siTRIM58-3	1454-1472	GGGCATCCAGGGATCATTT
